# Assessment of Renal Function in Patients with Unilateral Ureteral Obstruction Using Whole-Organ Perfusion Imaging with 320-Detector Row Computed Tomography

**DOI:** 10.1371/journal.pone.0122454

**Published:** 2015-04-15

**Authors:** Xiang-Ran Cai, Qing-Chun Zhou, Juan Yu, You-Zhen Feng, Zhao-Hui Xian, Wen-Cai Yang, Xu-Kai Mo

**Affiliations:** 1 Medical Imaging Center, First Affiliated Hospital, Jinan University, Guangzhou, Guangdong, P.R.China; 2 Department of Urology, Nanhua Affiliated Hospital, Nanhua University, Hengyang, Hunan, P.R.China; 3 Department of Radiology, Shenzhen Second People’s Hospital, Shenzhen, Guangdong, P.R.China; University of Bari Aldo Moro, ITALY

## Abstract

**Background:**

Obstructed nephropathy is a common complication of several disease processes. Accurate evaluation of the functional status of the obstructed kidney is important to achieve a good outcome. The purpose of this study was to investigate renal cortical and medullary perfusion changes associated with unilateral ureteral obstruction (UUO) using whole-organ perfusion imaging with 320-detector row computed tomography (CT).

**Methodology/Principle Findings:**

Sixty-four patients with UUO underwent whole-organ CT perfusion imaging. Patients were divided into 3 groups, mild, moderate, and severe, based on hydronephrosis severity. Twenty sex- and age-matched patients without renal disease, who referred to abdominal CT, were chosen as control subjects. Mean cortical and medullary perfusion parameters of obstructed and contralateral kidneys were compared, and mean perfusion ratios between obstructed and contralateral kidneys were calculated and compared. Mean cortical or medullary blood flow (BF) and blood volume (BV) of the obstructed kidneys in the moderate UUO and BF, BV, and clearance (CL) in the severe UUO were significantly lower than those of the contralateral kidneys (p < 0.05). The mean cortical or medullary BF of the obstructed kidney in the moderate UUO, and BF, BV, and CL in the severe UUO were significantly lower than those of the kidneys in control subjects (p < 0.05). Mean cortical or medullary BF of the non-obstructed kidneys in the severe UUO were statistically greater than that of normal kidneys in control subjects (p < 0.05). An inverse correlation was observed between cortical and medullary perfusion ratios and grades of hydronephosis (p < 0.01).

**Conclusions/Significance:**

Perfusion measurements of the whole kidney can be obtained with 320-detector row CT, and estimated perfusion ratios have potential for quantitatively evaluating UUO renal injury grades.

## Introduction

Obstructed nephropathy is a common complication of several disease processes. It usually causes progressive renal damage, and ultimately leads to renal failure. An accurate evaluation of the anatomical and functional status of the obstructed kidney is important to determine optimal management and assess the effectiveness of therapy, especially when the disease involves only one kidney.

Computed tomography (CT) perfusion imaging is a new, non-invasive technology that allows the quantification of renal blood flow abnormalities of a single kidney, as well as renal morphological changes [1–4]. Although other imaging modalities also allow tissue perfusion assessment, CT perfusion imaging has higher spatial and temporal resolution compared to isotope scintigraphy or magnetic resonance imaging (MRI). Another principal merit of CT perfusion is the linear relation between iodine concentration and the enhancement of the kidney, which simplifies mathematic calculation compared to MRI. Furthermore, the perfusion parameters can be acquired more easily with the application of perfusion software.

An innovative 320-detector row CT allows for 160 mm coverage in the z-direction without table movement, which makes it possible to perform volumetric perfusion. As such, perfusion information of the whole organ, such as bilateral kidneys, can be obtained. This CT system has been successfully used to evaluate the hemodynamics of liver, pancreas and spleen perfusions [5,6]. As to the urinary system, Zhong et al. [7] investigated perfusion changes of left kidneys in patients with nutcracker syndrome (NCS) using 320-detector volume CT. Chen et al. [8] investigated microcirculatory differences between pathological types of kidney tumor using the same CT system. However, perfusion studies based on this modern dynamic volume CT for the whole bilateral kidneys in patients with obstructive nephropathy have not been reported.

Thus, the purpose of this study was to determine the feasibility and effectiveness of whole-organ perfusion imaging based on 320-detector row dynamic volume CT for assessing renal hemodynamics and function in patients with unilateral ureteral obstruction (UUO).

## Materials and Methods

### Ethical considerations

This study was approved by the Committee for Medical Ethics, the First Affiliated Hospital of Jinan University, China, and was performed in accordance with the principles of the Declaration of Helsinki. Written informed consent was obtained from all patients.

### Patient population

Sixty-four consecutive patients with UUO as determined by ultrasound were enrolled in this study between January 2013 and September 2013. The patients included 25 females and 39 males with a mean age of 48.0 ± 4.5 years (range, 20–78 years). Thirty-one of the obstructed kidneys were on the right side, and 33 were on the left. The causes of UUO were ureteral stones in 61 patients, and ureteropelvic junction stricture in 3 patients. Patient symptoms included flank pain, hematuria, pyuria, and dysuria. Patients with renal failure (serum creatinine >1.5 mg/dl [114 mol/l]), bilateral obstruction, or unilateral obstruction with an abnormal or absent contralateral kidney, and underage patients (less than 18 years) were excluded. In order to assess the effect of the severity of obstruction on renal function, patients were classified into 3 groups according to their CT findings [9]. Mild was defined as flattening of the pelvis and major calyces, loss of waist, and mild ballooning with or without thinning of the renal parenchyma to 2.4–2.6 cm. Moderate was defined as moderate dilatation of the pelvis and ballooned calyces with thinning of the renal parenchyma to 1.8–2.3 cm. Severe was defined as a markedly dilated pelvis and calyces with marked thinning of the renal parenchyma to 0.6–1.5 cm. Prior to CT scan, all the subjects were asked to refrain from food and water for six hours.

A group of 20 sex- and age-matched individuals (mean age, 45.2 ± 7.6 years; range, 23–74 years) who had received upper abdominal perfusion imaging using 320-detector dynamic volume CT at our hospital were retrospectively chosen as control subjects. The subjects had no renal or renal vascular diseases or hypertension. All control subjects had normal serum creatinine levels, and they showed no morphologic abnormalities in both kidneys on CT examination.

### CT acquisition protocol

A low-dose perfusion protocol was used with a 320-detector row CT system (Aquilion ONE; Toshiba Medical Systems, Ohtawara, Japan). In order to minimize the radiation dose, we chose 100 kV as tube voltage, 100 mA as the mask tube current, and 60 mA as the following tube current. Other scan parameters were 0.5 mm × 320 collimator, 512 × 512 matrices, 320–350 mm field of view (FOV), and 0.5 s rotation time. Bilateral whole kidneys of each patient were covered by a single scan as a result of z-direction coverage of 160 mm.

A total of 56 ml anon-ionic contrast material (CM: Optiray, Ioversol Injection; 350 mg I/ml; Tyco Healthcare, Quebec, Canada) was injected with a flow rate of 7 ml/s using a dual-shot injector (Dual Shot Alpha; Nemoto-Kyorindo, Tokyo, Japan) through an 18-gauge intravenous injection catheter inserted into an antecubital vein. A 100 ml 0.9% saline chaser was administered with the same flow rate after the injection of CM. The dynamic volume CT scan was started at the 7^th^ second after the injection to obtain a mask, and acquisition was continued every 2 seconds intervals from the 12^th^ second to the 30^th^ second. Then, after a 3-second pause, 3-second intervals were used from the 33^th^ second to the 48^th^ second. Again, after a 7-second pause, 7-second intervals were used from the 55^th^ second to 90^th^ second. The patient was allowed to breathe gently throughout the examination.

The volumetric CT dose index (CTDIvol) and dose-length product (DLP) of each examination was automatically archived by the system when the scan was finished. The effective dose (ED) was calculated using an appropriate k-factor of 0.015 mSv/mGy·cm [10]. In order to reduce the toxic effect of the CM, patients were hydrated for 1 hour before contrast injection and for 6 hours after administration of the contrast agent. The initial intravenous saline bolus was 3 ml/kg, and subsequent fluid administration was 1 ml/kg per hour.

### Post-processing and data analysis

**Fig 1:**
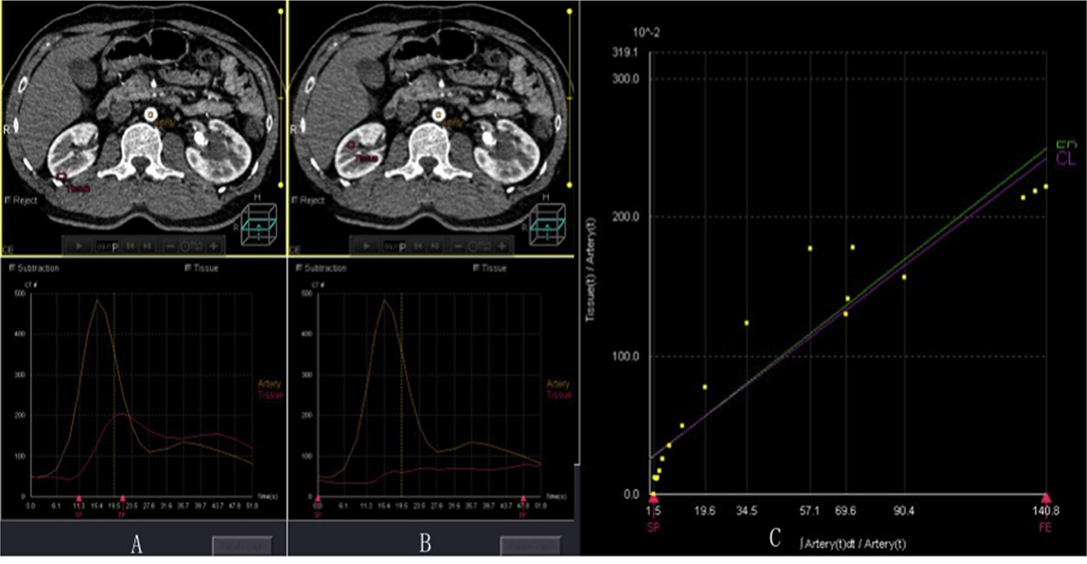
Time–density curves (TDCs) of renal cortex (a) and medulla (b) from the single input maximum slope and Patlak graph from the Patlak plot (c). TDC indicates the enhancement characteristics of the aorta and renal cortex or medulla during the first pass. BF can be determined from the maximum gradient of renal cortical or medullary TDC divided by the peak enhancement of the aorta. The Patlak plot is a graphical analysis method, which quantifies the passage of contrast from the intravascular space into the extravascular space. BV and CL can be obtained from the Patlak equation. TDC curve: SP: start phase; EP: end phase. Patlak graph: SP: start phase; FE: flow end; EP line: early perfusion regression line (green line); CL line: clearance regression line (purple line). BF, blood flow; BV, blood volume; CL, clearance.

Perfusion post-processing and measurement were performed by two experienced radiologists in consensus. The acquired perfusion volume data was loaded into the body perfusion software in the display console (Toshiba Medical System, Tochigi, Japan) for post-processing. First, volume registration was performed to eliminate the effect of breathing movement, and a new registered volume dataset was generated. Time–density curve (TDC) was obtained when regions-of-interest (ROIs) were placed on the abdominal aorta at the level of the renal hilum and renal cortex or medulla. A single input maximum slope algorithm was used to obtain a blood flow map (BF, ml/100 ml/min) based on TDC, and Patlak plot method was used to acquire a blood volume map (BV, ml/100 ml) and clearance map (CL, ml/100 ml/min) based on Patlak graph [8,11–13] (Fig 1). BF was defined as the flow rate through the vasculature in a given tissue region. BV indicated the volume of blood flowing within the vasculature in a given tissue region. CL was the total flux from plasma to the interstitial space.

**Fig 2:**
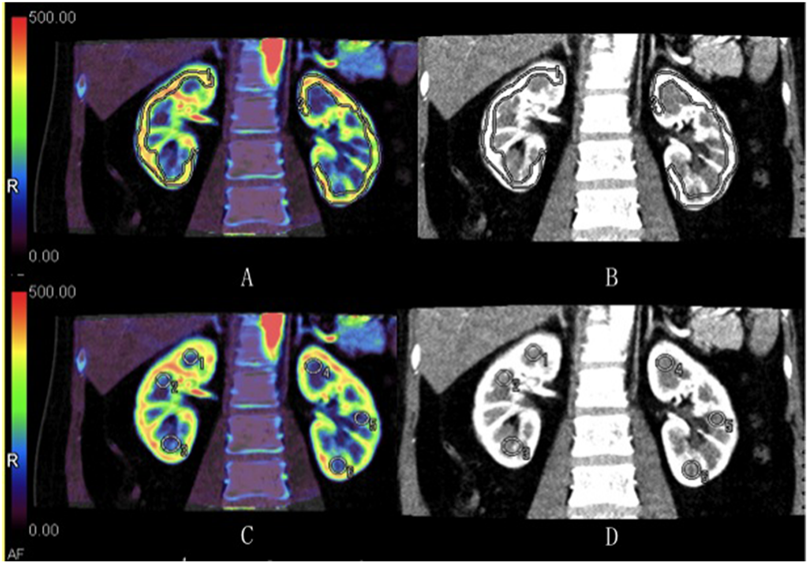
Examples for regions of interest (ROIs) measurements. ROIs of renal cortex (a, b) and medulla (c, d) were defined manually in the coronal plane.

ROIs for perfusion measurements were placed on the bilateral renal cortex and medulla on the perfusion maps and made as large as possible. ROIs of renal cortex and medulla were defined manually in axial and coronal planes for each area to minimize the influence of potential measurement errors (Fig 2). Moreover, ROIs were placed in three different sections of each of the two planes. A mean value was calculated, on which later statistical analysis was based. The ratios of BF, BV, and CL between the obstructed (or left for control subjects) and contralateral kidneys were then determined using the following formula: ratio = perfusion_obstructed or left_ / perfusion_contralateral_.

### Statistical analysis

The mean and standard deviation (mean ± SD) of measurements were calculated for all perfusion parameters. A paired sample t-test was used to compare data in the obstructed kidneys with those in the contralateral non-obstructed kidneys for UUO patients. A paired sample t-test was also used to compare data in the left kidneys with those in the right kidneys for control subjects. Analysis of variance (ANOVA) was used to assess the differences of the cortical or medullary perfusion values between UUO patients and control subjects. A Pearson correlation analysis was performed to examine the relationship between the perfusion values and the hydronephosis severity. A p value less than 0.05 was considered to indicate a significant difference. Statistical analysis was performed using SPSS version 16.0A (IBM, Armonk, NY, USA).

## Results

### Dose measurement and morphological evaluation

Examinations were performed successfully in all 64 patients without any technical problems or adverse reactions to the CM. The estimated mean ED was 11.29 ± 0.36 mSv (range, 10.98–11.77 mSv).

Dilatation of the collecting system in UUO patients was mild in 26 patients, moderate in 22 patients, and severe in 16 patients.

### Mean cortical and medullary perfusion parameters of bilateral kidneys in control subjects and UUO patients

**Fig 3:**
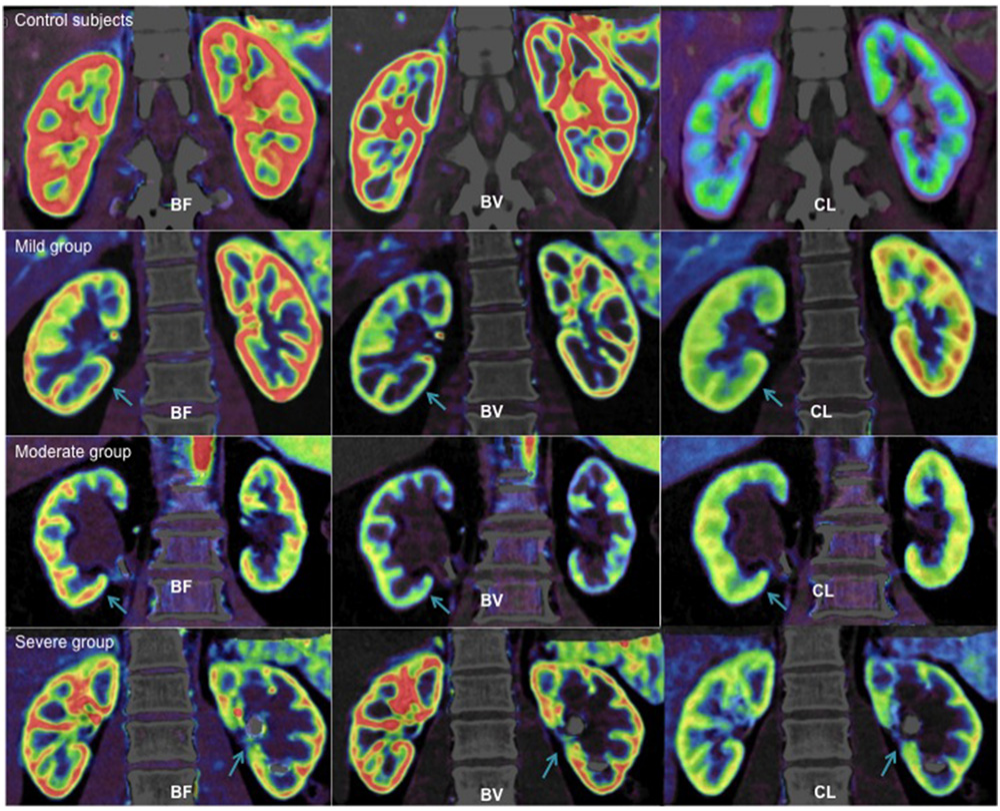
BF, BV and CL maps from control subjects and the three groups of UUO. The perfusions of both kidneys were uniform in control subjects and renal cortex showed an intact red ring. For UUO patients, BF, BV and CL maps showed dilation of collecting system in the obstructed kidney (arrow) and normal contralateral kidney. The renal cortex from obstructed kidney was less continuous and local yellow or blue areas were observed in the perfusion maps. BF, blood flow; BV, blood volume; CL, clearance.

The mean cortical and medullary perfusion parameters of bilateral kidneys in control subjects and UUO patients are shown in Table 1. The mean cortical or medullary BF, BV, and CL between the left and right kidneys were similar in control subjects. There were no statistically significant differences in any of these parameters. The mean cortical and medullary perfusion values of obstructed kidneys were lower than those of contralateral kidneys in UUO patients (Fig 3). In the cortex and medulla, the mean BF and BV in the moderate UUO, and BF, BV, and CL in the severe UUO were found to be significantly different between the obstructed and contralateral kidneys (p <0.05). However, no significant differences were observed in the cortical or medullary perfusion values of the mild UUO between the obstructed and contralateral kidneys (p >0.05).

**Table 1:** The mean cortical and medullary perfusion parameters of bilateral kidneys in control subjects and UUO patients.

	Control subjects	UUO		
		Mild group	Moderate group	Severe group
Left kidney (control subjects) or obstructed kidney (UUO)				
Cortical BF	266.15±63.38	251.18±73.11	189.54±58.81* ^#^	141.13±79.43* ^#^
Cortical BV	42.13±28.07	35.63±27.49	42.50±33.71*	16.29±6.62* ^#^
Cortical CL	11.74±4.13	11.16±2.92	9.06 ±4.22	6.11 ±3.31* ^#^
Medullary BF	118.64±36.88	121.75±32.42	83.86 ±26.48* ^#^	53.37±49.76* ^#^
Medullary BV	13.45±14.75	12.65±7.29	12.62±11.36*	8.45±9.75* ^#^
Medullary CL	10.93±4.71	9.67 ±4.39	7.83 ±3.99	6.05 ±4.17* ^#^
Contralateral kidney				
Cortical BF	268.68±58.77	276.2±60.65	239.3±62.82	334.85±130.91^#^
Cortical BV	42.87±27.36	38.02±22.80	49.12±31.99	41.28±16.16
Cortical CL	11.98±3.64	12.64±2.39	11.69±4.31	11.82±2.07
Medullary BF	115.48±38.78	124.47±29.40	109.36±14.53	132.02±45.69^#^
Medullary BV	13.27±12.53	13.11±10.12	15.34±14.88	10.90±5.26
Medullary CL	13.27±12.78	12.36±4.16	12.28±6.68	11.68±3.45

BF: ml/100 ml/min; BV: ml/100 ml; CL: ml/100 ml/min.

Abbreviation: UUO, unilateral ureteral obstruction; BF, blood flow; BV, blood volume; CL, clearance.

*Indicates p <0.05 by paired sample t-test when compared to contralateral kidney.

^#^Indicates p <0.05 by ANOVA when compared to the kidneys of control subjects.

Also, the mean cortical and medullary perfusion values of obstructed kidneys in UUO patients decreased as compared with kidneys of control subjects. In the cortex and medulla, the mean BF of obstructed kidneys in the moderate UUO, and BF, BV, and CL of obstructed kidneys in the severe UUO were significantly lower than those of the kidneys of control subjects (p <0.05). However, no significant differences in any perfusion values between them were observed in the cortex and medulla of the mild UUO (p >0.05). Mean cortical or medullary BF of non-obstructed kidneys in the severe UUO was statistically greater than that of normal kidneys in control subjects (p <0.05). However, no significant difference was found in the perfusion values between non-obstructed kidneys of the mild or moderate UUO patients and normal kidneys of control subjects (p >0.05).

### The mean cortical and medullary ratios of BF, BV, and CL between the obstructed and contralateral kidneys in UUO patients and control subjects

Mean cortical or medullary BF ratio, BV ratio, and CL ratio between the obstructed and contralateral kidneys in UUO patients and between the left and right kidneys in control subjects are shown in Table 2. Mean cortical BF ratio, medullary BF ratio and CL ratio in the moderate UUO were significantly lower than those of the kidneys of control subjects (p <0.05). The same results were also observed in the mean cortical or medullary BF ratio, BV ratio, and CL ratio between severe UUO and control subjects (p <0.05). No significant differences were found in the cortical or medullary any of these perfusion ratios between the mild UUO and control subjects (p >0.05).

**Table 2:** Mean cortical or medullary BF ratio, BV ratio, and CL ratio between the obstructed and contralateral kidneys in UUO patients and between the left and right kidneys in control subjects.

	Control subjects	UUO		
		Mild group	Moderate group	Severe group
Cortical BF ratio	0.99±0.07	0.91±0.20	0.79±0.14*	0.44±0.24*
Cortical BV ratio	0.98±0.21	0.90±0.27	0.79±0.24	0.51±0.12*
Cortical CL ratio	0.99±0.19	0.94±0.15	0.88±0.24	0.52±0.25*
Medullary BF ratio	1.05±0.21	0.92±0.22	0.77±0.26*	0.46±0.65*
Medullary BV ratio	1.00±0.25	1.18±0.32	0.62±0.22	0.55±0.17*
Medullary CL ratio	0.98±0.28	0.78±0.20	0.68±0.33*	0.33±0.47*

Abbreviation: UUO, unilateral ureteral obstruction; BF, blood flow; BV, blood volume; CL, clearance.

*Indicates p <0.05 by ANOVA when compared to control subjects.

In the cortex and medulla, the mean BF ratio, BV ratio, and CL ratio decreased with increasing the severity of hydronephrosis. Variations of the perfusion ratios across the mild, moderate, and severe groups were larger in the cortex than in the medulla. However, the same relationships were not observed with mean BF, BV, and CL values.

### Correlation analysis

Statistically significant correlations were detected between cortical or medullary BF ratio, BV ratio, and CL ratio and severity of hydronephrosis (cortical: r = -0.752, p <0.001; r = -0.518, p <0.001; r = -0.660, p <0.001, respectively; medullary: r = -0.514, p <0.001; r = -0.386, p <0.001; r = -0.494, p <0.001, respectively).

## Discussion

Electron-beam CT (EBCT) was first used to complete the perfusion imaging because of its high temporal resolution, which has been shown to provide reliable measurements of renal flow [14–16]. Multi-detector CT (MDCT) allows a rapid volume acquisition with a high spatial resolution, and thus has widespread application in clinical practice. Unlike EBCT, MDCT is currently available in all hospitals. A study by Daghini et al. [17] revealed that renal perfusion measurement with 64-slice MDCT was comparable to that of EBCT. Lemoine et al. [18] found a strong correlation between MDCT and fluorescent microspheres when measuring renal perfusion. Several previous studies demonstrated that MDCT perfusion was a useful method for renal functional evaluation [19–22]. However, the perfusion information in those early studies was obtained by sequential scans in a certain part of the kidney due to the limited coverage of detectors using conventional MDCT. Higher doses of radiation and more contrast agents were used because of more CT scans. Furthermore, the perfusion information from a single section could not represent the whole kidney. 320-detector row CT has been developed with a z-coverage value of 160 mm; thus, the whole bilateral kidneys can be scanned in a single rotation and volumetric perfusion information can be easily obtained.

In the current study, 320-detector row dynamic volume CT successfully assessed the bilateral function through the volumetric perfusion imaging, reflecting the intra-renal hemodynamic changes of both the obstructed and contralateral kidneys in UUO patients. Preoperative perfusion information of obstructed kidney is one of the independent factors affecting renal function recovery in patients with UUO. Quantitative assessment of relative individual renal function can help the urologists track the effectiveness of treatment and influence the clinical judgment. Compared with other imaging modalities, CT perfusion is relatively low-cost, fast and safe, especially on an advanced 320-dectector row CT with a well-refined protocol. The ED in this study was 11.29 mSv, which was lower than that reported in some previous studies with the same CT system [5,6,8].

Our study results demonstrated a decrease in the perfusion parameters of the obstructed kidneys, suggesting the abnormal low perfusion in the cortex and medulla in patients with a moderate and severe unilateral ureteral obstruction. This finding is supported histological changes seen after the ureteral obstruction. The early histological patterns include increasing edema in Bowman’s space, tubular dilatation, flattening and atrophy, and peritubular inflammation. Long existence of obstruction can lead to high risks of glomerular sclerosis, reduction of renal glomeruli, and interstitial fibrosis [23–27]. Furthermore, many animal studies indicated that enhancement of the renin-angiotensin system (RAS) and insufficient nitric oxide synthesis led to vasoconstriction in an obstructed kidney resulting in a reduction of renal blood flow [28]. This fall in renal flow is similar to those previously reported in other studies [14,24,29,30]. In the current study, a slight decrease in the perfusion values was noted in the mild UUO, but it was not statistically significant. This suggests that slight dilation of pelvis and main calyx is not enough to cause significant perfusion decline of the renal cortex and medulla.

In the cortex and medulla, mean BF value of non-obstructed kidneys in the severe UUO was statistically greater than that of normal kidneys in control subjects. However, no significant difference was found in all of hemodynamic parameters between non-obstructed kidneys of the mild or moderate UUO patients and normal kidneys of control subjects. Similarly, Lerman et al. [16] reported BF increased in the contralateral kidney after a month of hypertension created by unilateral renal artery stenosis in pigs. This might be explained by a compensatory increase of BF in the contralateral kidney because of severe impaired function in the obstructed kidney. However, in the case of mild and moderate obstruction, there was no significant compensatory increase in BF. This finding suggested that compensatory growth of the contralateral kidney is mainly related to the degree and rate of functional deterioration in the obstructed kidney. In contrast, in animal models with acute UUO [25,29], no hemodynamic changes of the contralateral kidney were found, possibly reflecting differences between acute and chronic UUO. Another potential factor leading to this difference might be individual differences in the perfusion values. The kidney is the organ with highest blood flow in the body, and it receives approximately a quarter of the cardiac output. This means that renal blood flow varies in each individual. It is evident that in some patients the cortical and medullary perfusion values of the obstructed kidney are higher than in the healthy kidney of the other patients and control subjects in our study. The differences in the perfusion values are mainly attributed to different physiological conditions of the patients. Similar to our results, Zhong et al. [7] found that the renal cortex perfusion of Nutcracker patients differed from each other in different individuals. Thus, it is inadvisable to evaluate the renal function injury of one patient based on perfusion values in different individuals.

In our study, average BF values of left and right renal cortex in healthy control group were 266.15 and 268.68 ml/100ml/min, which were different from those reported in previous studies with the same CT system [7,8]. Zhong et al. [7] reported that the BF values of left and right renal cortex were 323.8 and 322.9 ml/100ml/min in control group. And Chen et al. [8] reported that the BF value of normal renal cortex was 305.4 ml/100ml/min. A possible explanation for this discrepancy could be differences in age distribution, scan protocols and study population. Nonetheless, the medullary BF values were not measured in previous studies. Furthermore, BF values in UUO could not be compared with previous studies [14,19,29,30] because of different CT system, perfusion post-processing algorithms and research subjects.

In the current study, there were no significant differences in mean cortical or medullary perfusion values between the left and right kidneys in control subjects. This indicates that BF between the left and right kidneys in the healthy subjects is uniform. Therefore, it might be more reasonable to evaluate renal damage of one side using perfusion values of the other side as the reference. We also found that mean cortical and medullary perfusion ratios between obstructed and contralateral kidneys decreased with increasing severity of hydronephrosis. Also, inverse correlations were observed between perfusion ratios and hydronephrosis grades. These results are consistent with those reported by Pelaez et al. [29] and Sheehan et al. [30]. Taken together, these findings support that the perfusion ratios can reflect the haemodynamic changes and renal functional damage grades in UUO patients. However, it should be noted that in severe UUO, the perfusion ratios might be artificially lowered due to the compensatory increase in BF in the contralateral kidney.

The present study has several limitations. First, the overall number of patients was small. A larger number of patients may provide more precise values. Second, although the low-dose perfusion protocol used in the current study allows lowering the tube current-time product to 30 mAs, the radiation dosage for CT perfusion imaging is still of great concern. Liu et al. [31] achieved one-tenth dose CT perfusion images (80 kV, 16 mAs) without loss of accuracy by using the highly constrained back-projection (HYPR)-local reconstruction (LR) noise-reduction technique. Consequently, further reduction of radiation dose may be achieved with such a technique. Third, patients undergoing CT perfusion imaging are inevitably exposed to iodinated CM, which can potentially result in contrast-induced nephropathy (CIN). However, the amount of CM used in the current study was small. Many studies have demonstrated that the risk of CIN is highly associated with the volume of CM used [32,33]. Moreover, patients were hydrated to reduce the possibility of CIN.

## Conclusions

This study showed that 320-detector row dynamic volume CT is capable of detecting the hypoperfusion of an obstructed kidney as compared to a healthy kidney. CT perfusion imaging is a simple, accurate, and non-invasive technique that allows quantified assessment of the function of a single kidney affected by UUO as determined by significantly different perfusion values from that of a healthy kidney. Estimated perfusion ratio values between the obstructed kidney and the healthy kidney have the potential for quantitatively evaluating UUO renal injury grades.
